# Thermal sensitivity of growth indicates heritable variation in 1-year-old rainbow trout (*Oncorhynchus mykiss*)

**DOI:** 10.1186/s12711-016-0272-3

**Published:** 2016-11-29

**Authors:** Matti Janhunen, Juha Koskela, Nguyễn Hữu Ninh, Harri Vehviläinen, Heikki Koskinen, Antti Nousiainen, Ngô Phú Thỏa

**Affiliations:** 1 Biometrical Genetics, Natural Resources Institute Finland (Luke), Myllytie 1, 31600 Jokioinen, Finland; 2 Aquaculture, Natural Resources Institute Finland (Luke), Survontie 9 A, 40500 Jyväskylä, Finland; 3 Research Institute for Aquaculture No. 3 (RIA-3), Nha Trang, Khanh Hoa Vietnam; 4 Tervo Fish Farm, Natural Resources Institute Finland (Luke), Huuhtajantie 160, 72210 Tervo, Finland; 5 Research Institute for Aquaculture No. 1 (RIA-1), Dinh Bang, Tu Son, Bac Ninh Vietnam

## Abstract

**Background:**

Rainbow trout is an important aquaculture species, which has a worldwide distribution across various production environments. The diverse locations of trout farms involve remarkable variation in environmental factors such as water temperature, which is of major importance for the performance of fish. Thus, robust fish that could thrive under different and suboptimal thermal conditions is a desirable goal for trout breeding. Using a split-family experimental design (40 full-/half-sib groups) for a rainbow trout population derived from the Finnish national breeding program, we studied how two different rearing temperatures (14 and 20 °C) affect feed intake, growth rate and feed conversion ratio in 1-year-old fish. Furthermore, we quantified the additive genetic (co-)variation for daily growth coefficient (DGC) and its thermal sensitivity (TS), defined as the slope of the growth reaction norm between the two temperatures.

**Results:**

The fish showed consistently lower feed intake, faster growth and better feed conversion ratio at the lower temperature. Heritability of TS of DGC was moderate ($h_{\text{TS}}^{2} = 0.24$). The co-heritability parameter derived from selection index theory, which describes the heritable variance of TS, was negative when the intercept was placed at the lower temperature (−0.28). This resulted in moderate accuracy of selection. At the higher temperature, co-heritability of TS was positive (0.20). The genetic correlation between DGC and its TS was strongly negative (−0.64) when the intercept was at the lower temperature and positive (0.38) but not significantly different from zero at the higher temperature.

**Conclusions:**

The considerable amount of genetic variation in TS of growth indicates a potential for selection response and thus for targeted genetic improvement in TS. The negative genetic correlation between DGC and its TS suggests that selection for high growth rate at the lower temperature will result in more temperature-sensitive fish. Instead, the correlated response of TS is less pronounced if the selection for a higher DGC occurred at the higher temperature. It seems possible to control the correlated genetic change of TS while selecting for fast growth across environments, especially if measurements from both environments are available and breeding values for reaction norm slope are directly included in the selection index.

**Electronic supplementary material:**

The online version of this article (doi:10.1186/s12711-016-0272-3) contains supplementary material, which is available to authorized users.

## Background

Different genotypes, which are typically referred to as sib-groups, strains or populations, may differ in their average performance response to environmental variables. In wild populations, an organism’s ability to modify its phenotype in response to environmental changes (termed phenotypic plasticity) can itself be an adaptive life-history trait, which is subject to natural selection [1–4]. Phenotypic plasticity is considered synonymous to macro-environmental sensitivity, which is a more commonly used term in the animal breeding context [5]. For animal breeders, macro-environmental sensitivity is an important aspect due to its association with the animals’ performance across production environments, and with their robustness (stability) and welfare [6–9].

For any measurable phenotypic trait, the macro-environmental sensitivity of a genotype can be illustrated as the response function with environmental change [10]. Assuming a linear reaction norm, the degree of sensitivity for a genotype can be quantified by the regression slope of a genotype’s performance across an environmental gradient [11, 12]. The existence of macro-environmental sensitivity for a given trait is indicated by slopes that deviate from zero, whereas flat reaction norms across the environmental gradient axis reflect stability of the trait. Since the reaction norms also depict the extent of re-ranking among genotypes and the change in additive genetic variance with the environment (i.e., two forms of genotype × environment interaction) they can provide information about the capacity of populations and species to adapt to environmental variability [13].

The rainbow trout, *Oncorhynchus mykiss* (Walbaum), is an example of a globally important aquaculture species, which is distributed across various production environments and systems, which range from offshore net cages to land-based re-circulation facilities. The diverse geographical locations of rainbow trout farms may involve considerable variation in many abiotic (e.g., water temperature, salinity, and photoperiod) and biotic (quality of feed, pathogens and parasites) factors, which are of major importance for the performance of fish. High growth capacity may be considered worldwide as the single most economically important trait to be improved by selective breeding [14], but the capacity of fish to express the selected growth potential under variable or suboptimal environmental conditions may be constrained. Therefore, a more robust fish material with stabile growth would be an eligible product for breeding under variable environmental conditions.

Being native to cool, temperate regions of the northern hemisphere, the rainbow trout, like all other salmonids, is adapted to relatively low water temperatures [15, 16]. Stability of growth is of special importance in trout farming areas, where rearing temperatures remain constantly high or where strong seasonal warming occurs. Furthermore, due to global warming, it is likely that there will be an increasing demand in the fish farming sector for populations of more heat-tolerant trout in the future. To assess whether thermal sensitivity (TS) of growth has the potential to be changed by selection, an estimate of the additive genetic component in the slopes of reaction norms is needed. The existence of genetic variation in growth responses as the temperature changes would enable the development of more temperature-tolerant or locally-adapted populations for different thermal conditions.

In this study, we first investigated at a general (population) level how two different rearing temperatures (14 °C, namely ‘low’, and 20 °C, namely ‘high’) influence feed intake, growth and feed conversion ratio in 1-year-old rainbow trout. Second, by using a split-family design of the experiment, we quantified the additive genetic (co-)variation of growth rate (daily growth coefficient, DGC) and its TS, the latter trait being defined as the slope of the reaction norm between the two temperature conditions.

## Methods

### Study material

The fish used in this study were derived from the Finnish national breeding program that is maintained at the Tervo fish farm (breeding nucleus) by the Natural Resource Institute Finland (Luke). The phenotypic data comprised 800 individuals from 40 families, which were created in April 2013 using a partial factorial mating design for 35 sires and 26 dams. Each sire was mated to an average of 1.1 dams (ranging from 1 to 3) and each dam to an average of 1.5 sires (ranging from 1 to 3). The average number of offspring was 22.9 per sire (ranging from 20 to 60) and 30.8 per dam (ranging from 20 to 60). The parental fish were selected using a multi-trait selection index with the main weight on improved growth (50% of the index). The pedigree file included 1661 individuals and nine generations tracing back to the base population established in 1989 and 1990 (see Additional file 1).

### Rearing protocol

In this study, the protocols used were approved by the FGFRI Animal Care Committee, Helsinki, Finland.

The first 6 months of rearing took place in the breeding nucleus, where the full-sib families were reared separately in round 150-L indoor tanks until tagging. Variation in rearing temperature followed variation in ambient waterway throughout that period (ranging from 0 to 20 °C from the start of hatching until the start of id-tagging). During the period between January 10 and 21 2014, 25 randomly chosen fish from each studied family (73.6 ± 14.4 g, mean weight ± SD) were individually tagged with passive integrated transponders (Biomark, Inc., Boise, Idaho, USA). The tagged fish were transported into the communal pool at the Laukaa fish farm, where they were reared under ambient temperature (1–10 °C) and light conditions (day length 16.30–21.30 h during the last month prior to the experiment) until start of the experiment. In June 2014, 20 tagged fish per family were randomly sampled for the temperature trial. The rearing temperatures were gradually increased to the experimental temperatures (14 and 20 °C) in the course of 3 days. To construct a split-family design, each of the 40 families was first randomly split into two groups to be reared at low and high temperatures. These groups were evenly distributed over 4 + 4 (low temperature) and 4 + 4 (high temperature) round 0.4-m^3^ green plastic tanks (two replicate tanks per family). In total, the temperature trial began with a total of 800 fish, each tank containing 50 fish (ten families and five fish from each family).

The trial was conducted from June 3 to August 12 2014. The fish were fed ad libitum 6 h per day (4.00 am to 10.00 am) using belt feeders with commercial trout diet (Raisioagro Ltd, Finland Vital pro LP; chemical composition given by the manufacturer 3.5/5.0 mm; crude protein 43.0/40.0%, crude fat 28.0/30.0%, crude fibre 1.5/1.5%, ash 6.5/6% and gross energy 24.4/24 MJ kg^−1^). From days 1 to 10, the fish were fed with 3.5-mm pellets, followed by a mixture (1:1) of 3.5- and 5.0-mm pellets from day 11 to 20, and thereafter with 5.0-mm pellets until the end of trial. During the experiment, daily feeding amount was increased so that the share of waste feed ranged from 0 to 35% of the level of daily feed. The numbers of uneaten pellets were collected at the tank outlet in a box with a mesh bottom. The daily number of waste pellets was calculated, and their weight was estimated by multiplying the number of waste pellets by the air dry weight of a pellet. Before calculations, five 100-pellet subsamples were taken from each of the diets to measure air dry weight of pellets. The daily intake of a tank’s population was calculated as the difference in weight between the fed and waste feed. Tanks of the low-temperature group were supplied with fresh lake water and tanks of the high-temperature groups were supplied with water via semi-intensive recirculating aquaculture systems (RAS; flow rate of makeup water 4–6 m^3^ kg^−1^ feed). Pure oxygen was added to incoming water for both temperature groups to improve water oxygen content and baking soda was added to the RAS to maintain the pH between 6.7 and 7.0.

During the experiment, the water temperature was automatically recorded hourly (low-temperature group 14.1 ± 1.0 °C and high-temperature group 20.4 ± 1.8 °C; mean ± SD). Water oxygen saturation (%) was recorded once every second week (low-temperature/high-temperature; tank inlet: 96.5 ± 7.8/105.7 ± 4.9, tank outlet: 78.6 ± 6.8–83.3 ± 7.1/82.0 ± 3.5–86.8 ± 5.2) and other water quality parameters were recorded weekly (high temperature pH 6.9 ± 0.1, total ammonia mg L^−1^ (NH_3_ + NH_4_) 0.05 ± 0.04, un-ionized ammonia mg L^−1^ (NH_3_) <0.001, nitrite mg L^−1^ (NO_2_) 0.07 ± 0.01, nitrate mg L^−1^ (NO_3_) 4.45 ± 0.7). A 24-h white light was provided with led lamps on the tank cover.

### Measurements and calculations

Individual tag number and body weight (to the nearest g) were recorded at the beginning (129 ± 28 g; mean ± SD, *n* = 800 fish) and end of the trial (516 ± 98 g, *n* = 785). Fifteen fish died during the trial, thus only the initial body weight was available for these. Daily growth coefficients (DGC, % day^−1^) of 785 fish were calculated as [17]:

$${\text{DGC}} = \left[ {\left( {{\text{BW}}_{2}^{1/3} - {\text{BW}}_{1}^{1/3} } \right)/{\text{t}}} \right] \times 100,$$

where BW_1_ and BW_2_ are the body weight of the fish at the start and end of the experiment, and t is the duration of the experiment (69–70 days) (see Additional file 2). Unlike specific growth rate (SGR), another widely used measure of fish growth rate, the DGC is independent of fish body weight and time interval between weighings at a given temperature [17]. This was also validated for rainbow trout [18].

The mean feed intake of a tank’s population per day (FI_mean_, g day^−1^) was calculated as FI_cum/_t1, where FI_cum_ is cumulative feed intake (g) of a tank’s population during the period of feed intake measurements divided by the number of measurement days (t1 = 57d). The relative feed intake (FI % biomass^−1^ day^−1^) was calculated as follows: $100 \times {\text{FI}}_{\text{mean}} /\left[ {\left( {{\text{biomass}}_{1} + {\text{biomass}}_{2} } \right)/2} \right]$, where biomass_1_ and biomass_2_ are the initial and final tank biomasses (g), respectively (see Additional file 3). Feed conversion ratio (FCR) was calculated as ${\text{FI}}_{\text{mean}} \times {\text{t}}/\left( {{\text{biomass}}_{2} - {\text{biomass}}_{1} } \right)$.

### Statistical analyses

The difference in DGC means between temperature treatments was tested for individual data using restricted maximum likelihood method in SAS^®^ 9.4 (MIXED procedure; SAS^®^ Institute, Cary, NC, USA). The model used was:

1 $$y_{ijk} = {\text{treatment}}_{j} + {\text{tank}}\left( {\text{treatment}} \right)_{k} + e_{ijk} ,$$

where *y* is the observation of the *i*th individual, treatment_*j*_ is the fixed effect of temperature treatment (*j* = 1–2), tank_*j*_ is the random effect of rearing tank during the trial (*k* = 1–16), nested within treatment, and *e*_*ijk*_ is the random error term. Error variances were modelled separately for each temperature condition. In addition, degrees of freedom for the test of the fixed effect were corrected using the method of Kenward and Roger [19].

For the relative feed intake and FCR, tank population values were used as observations (*n* = 8 per treatment group) and the analysis of variance (ANOVA) was used to compare the differences in means between the treatment groups. No covariate was used in these models.

Genetic (co)variance of TS (regression slope) for DGC was estimated using a linear random regression model (also termed as a reaction norm model). (Co)variance components were estimated by restricted maximum likelihood in ASReml 3.0 [20]. Approximate standard errors were calculated with ASReml according to Fisher et al. [21]. The linear random regression model was as follows:

2 $$y_{hij} = \beta_{\text{int}} + \beta_{\text{sl}} X_{\text{h}} + a_{{i,{\text{int}}}} + a_{{i,{\text{sl}}}} X_{h} + e_{hij} ,$$

where $\beta_{\text{int}}$ is the fixed regression coefficient for the population intercept (int) and $\beta_{\text{sl}}$ is the overall fixed regression slope (sl) of the trait on the *h*-th levels of an environmental gradient *X*_*h*_. *X*_*h*_ is the regressor for the environments in which the intercept was placed either on the low or high temperature environment (at *X*_*h*_ = 0). The value of *X*_*h*_ was 6 for high temperature and −6 for low temperature when these environments were not used at the intercept. The scale of *X* is equivalent to the difference in experimental temperatures, applying a unit of 1 °C. The values of 0 and 6 (or −6) were used instead of the actual temperature values (14 and 20 °C) in order to have an appropriate interpretation for the intercept. *a*_*i*_ is the random genetic effect of the intercept and slope of reaction norm, $$\left[ {\begin{array}{*{20}c} {a, {\text{int}}} \\ {a, {\text{sl}}} \\ \end{array} } \right]\sim{\text{MVN}}\left[ {\mathbf{0},{\mathbf{A}} \otimes {\mathbf{G}}} \right]$$, where MVN is a multivariate normal distribution, ${\mathbf{A}}$ is the additive genetic relationship matrix derived from the pedigree traced back to the base population, and ${\mathbf{G}}$ is the additive genetic covariance matrix: $${\mathbf{G}} = \left[ {\begin{array}{*{20}c} {\sigma_{{a,{\text{int}}}}^{2} } & {\sigma_{{a,{\text{int}},{\text{sl}}}} } \\ {\sigma_{{a,{\text{int}},{\text{sl}}}} } & {\sigma_{{a,{\text{sl}}}}^{2} } \\ \end{array} } \right]$$, where $\sigma_{{a,{\text{int}}}}^{2}$ and $\sigma_{{a,{\text{sl}}}}^{2}$ are the additive genetic variances of the intercept and slope, respectively, and $\sigma_{{a,{\text{int}},{\text{sl}}}}$ is the additive genetic covariance between the intercept and slope. $$e \sim N\left( {\mathbf{0},\left[ {\begin{array}{*{20}c} {{\mathbf{I}}\sigma_{{e_{1} }}^{2} } & 0 \\ 0 & {{\mathbf{I}}\sigma_{{e_{2} }}^{2} } \\ \end{array} } \right]} \right)$$ is the random residual effect of individual *i* in environment *h* where ${\mathbf{I}}$ is the identity matrix with a different residual variance for each environment. In addition, the random term ${\text{tank}}_{k} \times {\text{fullsib}}_{l}$, accounting for the interaction effect of experimental tank and full-sib family (modelled without the effect on the slope; *k* = 1–8 at low temperature and 11–18 at high temperature, *l* = 1–40), was tested. This variance parameter took the permanent environment effects into account, which were caused by different rearing tanks between and within families. However, based on the likelihood ratio test, inclusion of the ${\text{tank}}_{k} \times {\text{fullsib}}_{l}$ term did not affect the fit of the model (*χ* ^2^ = 0.001, *p* = 0.486). Therefore, the results are only presented for the models in which this variance parameter was omitted.

Both the magnitude and sign of a genetic correlation between the intercept and slope, as well as the genetic variance of the trait, depend on the environment for which the intercept is defined [22]. Therefore, the random regression model was run twice, either with low $(X_{{h_{1} }} = 0)$ or high temperature treatment $(X_{{h_{2} }} = 0)$ as the intercept environment. The covariance between estimated breeding values (EBV) of DGC at intercept (int) and slope (sl) was graphically illustrated by choosing five sires with the highest absolute EBV (i.e., genetically the most sensitive sires) and another five sires with close to zero EBV for the slope (the least sensitive) for drawing the reaction norms. Since there were only two environments, the EBV of DGC for the high temperature environment could be derived from a simple equation:

3 $${\text{EBV}}_{{{\text{DGC}},{\text{highT}}}} = {\text{EBV}}_{{{\text{int}},{\text{lowT}}}} + 6 \times {\text{EBV}}_{\text{sl}} .$$

The genetic values of the slope are multiplied by 6 to adjust the reaction norms to the scale of *X* (change of 6 °C).

For the intercept of the reaction norms, heritability $(h_{\text{int}}^{2} )$ was calculated as: $h_{\text{int}}^{2} = \widehat{\sigma }_{{a,{\text{int}}}}^{2} /\widehat{\sigma }_{{P,{\text{int}}}}^{2}$, where $\widehat{\sigma }_{{P,{\text{int}}}}^{2}$ is the phenotypic variance of DGC in the intercept environment when *X* = 0 (equal to the sum of additive genetic and residual variance in the intercept environment: $\widehat{\sigma }_{{a,{\text{int}}}}^{2} + \widehat{\sigma }_{{e,{\text{int}}}}^{2}$).

Because there is no phenotypic variance for the slope, the strict sense heritability cannot be calculated. Therefore, two alternative parameters were used to describe the genetic characteristics of TS. Following Sae-Lim et al. [23], the heritability for TS ($h_{\text{TS}}^{2}$) was calculated as:

4 $$h_{\text{TS}}^{2} = \frac{{\widehat{\sigma }_{{a,{\text{sl}}}}^{2} \times \widehat{\sigma }_{a,X}^{2} }}{{\widehat{\sigma }_{{P,{\text{Total}}}}^{2} }} ,$$

where $\widehat{\sigma }_{{a,{\text{sl}}}}^{2} \times \widehat{\sigma }_{a,X}^{2}$ is the additive genetic variance of the slope multiplied by the variance of *X*, respectively. $\widehat{\sigma }_{X}^{2}$ is equal to 18 in this study, since the values of *X* are 0 and 6. The standardized numerator in Eq. (4) is equivalent to the variance of the genotype by environment (GxE) interaction, which is independent from the different scales of an environmental variable $X$. The denominator $\widehat{\sigma }_{{P,{\text{Total}}}}^{2}$ was defined as follows:

$$\widehat{\sigma }_{{P,{\text{Total}}}}^{2} = \left[ {\frac{{\left( {n_{\text{lowT}} - 1} \right)\widehat{\sigma }_{{P_{{{\text{DGC}},{\text{lowT}}}} }}^{2} + \left( {{\text{n}}_{\text{highT}} - 1} \right)\widehat{\sigma }_{{P_{{{\text{DGC}},{\text{highT}}}} }}^{2} + n_{\text{lowT}} n_{\text{highT}} \left( {\overline{G}_{\text{lowT}} - \overline{G}_{\text{highT}} } \right)^{2} /n_{\text{lowT}} + n_{\text{highT}} }}{{n_{\text{lowT}} + n_{\text{highT}} - 1}}} \right],$$

where $n$ is the number of individuals with a record for an animal trait, $\widehat{\sigma }_{{P_{\text{DGC}} }}^{2}$ is the estimated phenotypic variance of the trait and $\overline{G}$ is the raw phenotypic mean of DGC. The denominator was derived from Scheiner’s approach, where the total phenotypic variance across environments was calculated from an analysis of variance [24]. However, it is important to note that $\widehat{\sigma }_{{P,{\text{Total}}}}^{2}$ is not the phenotypic variance of TS, and thus $h_{\text{TS}}^{2}$ is a descriptive rather than a predictive parameter [24]. The definition of heritability in Eq. (4) does not correspond with the conventional definition of heritability, which is the regression of breeding value on phenotype.

Following Sae-Lim et al. [23], an alternative measure that is called co-heritability was defined on the basis of selection index principles. Co-heritability is expressed as the regression coefficient (*b*) of the breeding value of slope on the phenotype *P* and defines the heritable genetic variance of TS of DGC when the selection criterion is DGC in one environment. The phenotypic variance ($\sigma_{P}^{2}$) of a trait is:

5 $$\sigma_{P}^{2} = \sigma_{{a,{\text{int}}}}^{2} + 2X\sigma_{{a,{\text{int}},{\text{sl}}}} + X^{2} \sigma_{{a,{\text{sl}}}}^{2} + \sigma_{e}^{2} ,$$

Co-heritability can vary in magnitude and have both positive and negative values, depending on which environment is set as intercept environment (*X* = 0). The sign of the co-heritability explains the change in correlated response of TS when mass selection for higher phenotypic values of DGC is practiced in one environment [25]. Here, the selection on DGC was assumed to be performed either at low or high temperature (intercept environment; *X* = 0), which gives the co-heritability *b* in the following equation:

6 $$b = \frac{{6\widehat{\sigma }_{{a,{\text{int}},{\text{sl}}}} }}{{\widehat{\sigma }_{{a,{\text{int}}}}^{2} + \widehat{\sigma }_{{e,{\text{int}}}}^{2} }} = \frac{{6\widehat{\sigma }_{{a,{\text{int}},{\text{sl}}}} }}{{\widehat{\sigma }_{{P_{h} }}^{2} }},$$

where $\widehat{\sigma }_{{P_{h} }}^{2}$ is the phenotypic variance of DGC in the selection environment *h*. Co-heritability is predictive for response to selection.

The genetic correlation between intercept and slope $\left( {r_{{G\left( {{\text{int}},{\text{sl}}} \right)}} } \right)$ was calculated as:

7 $$r_{{G\left( {{\text{int}},{\text{sl}}} \right)}} = \frac{{\widehat{\sigma }_{{a_{{{\text{int}},{\text{sl}}}} }} }}{{\sqrt {\widehat{\sigma }_{{a,{\text{int}}}}^{2} \times \widehat{\sigma }_{{a,{\text{sl}}}}^{2} } }} .$$

Finally, the accuracy (*r*_IH_) of EBV for TS when DGC is used as a selection criterion in one of the environments is equal to:

8 $$r_{\text{IH}} = \frac{{\widehat{\sigma }_{{a,{\text{int}},{\text{sl}}}} + X\widehat{\sigma }_{{a,{\text{sl}}}}^{2} }}{{\widehat{\sigma }_{P} \widehat{\sigma }_{{a,{\text{sl}}}} }} ,$$

where $\sigma_{P}$ is the phenotypic standard deviation and $\sigma_{{a, {\text{sl}}}}$ is the slope standard deviation. Equation (4) is equivalent to that derived by Kolmodin and Bijma [25].

## Results

### Means of growth rate, feed intake and feed conversion ratio

**Fig. 1:**
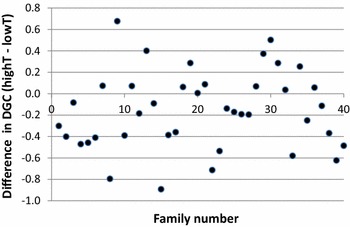
Difference in daily growth coefficient means for rainbow trout families reared at low (14.1 °C) and high (20.4 °C) temperatures. Families with a negative value grew better at low than at high temperature

The low-temperature group had slightly but significantly higher DGC means than the high-temperature group (Table 1). For 25 of the 40 families, the raw (uncorrected) DGC mean was higher at the lower temperature (Fig. 1). In contrast, both feed intake (FI) and feed conversion ratio (FCR) means were significantly lower in the low-temperature group (Table 1).

**Table 1:** Effect of rearing temperature (lowT 14.1 °C and highT 20.4 °C) on daily growth coefficient (DGC), feed intake (FI) and feed conversion ratio (FCR, intake/gain)

Treatment	DGC % day^−1^	FI % biomass^−1^ day^−1^	FCR
LowT	4.35 ± 0.63	1.66 ± 0.05	0.88 ± 0.01
HighT	4.19 ± 0.53	1.74 ± 0.05	0.97 ± 0.02
*F*-ratio	5.13	8.40	115.9
*df*	1, 13.5	1, 14	1, 14
*p* value	0.041	0.012	< 0.001

*df* = degrees of freedom

The values are raw phenotypic mean ± S.D. DGC was analysed using data from individual fish, whereas tank values were used as observations for FI and FCR

### Genetic (co)variance of DGC and its thermal sensitivity

The heritability of the slope that defines TS was moderate ($h_{\text{TS}}^{2}$ = 0.24), which indicates that the additive genetic variation in TS constitutes quite a large proportion of the total phenotypic variation in DGC across environments. The co-heritability for TS was moderately negative (−0.28) when the lower temperature was assigned as the intercept environment. This indicates that a moderate accuracy of selection for TS of growth can be achieved when individual selection for DGC is practiced in a low-temperature environment (*r*_IH_ = 0.44). Instead, the co-heritability estimate was positive (0.20) when a high-temperature environment was used for the intercept. This resulted in lower accuracy (0.25), compared to that of selection at the lower temperature. For the DGC at the intercept, the heritability estimates were similar and moderate ($h_{\text{int}}^{2}$ = 0.46) at both temperatures (Table 2). Thus, the heritable potential for growth rate did not change between the two temperature conditions.

**Table 2:** Genetic parameters and genetic correlations (±their approximate standard error) between intercept and slope obtained from the random regression models for daily growth coefficient (DGC) when the intercept was placed either in the low or high temperature environment

Parameter	Intercept environment	
	LowT	HighT
$\widehat{\sigma }_{{a, {\text{int}}}}^{2}$	0.190 (0.063)	0.130 (0.043)
$\widehat{\sigma }_{{e, {\text{int}}}}^{2}$	0.221 (0.041)	0.156 (0.028)
$\widehat{\sigma }_{{a, {\text{sl}}}}^{2}$	0.005 (0.002)	0.005 (0.002)
$\widehat{\sigma }_{{P, {\text{Total}}}}^{2}$	0.354	0.354
$h_{\text{int}}^{2}$	0.463 (0.123)	0.455 (0.120)
$h_{\text{TS}}^{2}$	0.244	0.244
Co-heritability of TS	−0.284 (0.124)	0.197 (0.141)
$r_{{{\text{G}}({\text{int}},{\text{sl}})}}$	−0.643* (0.147)	0.376 (0.214)

$\widehat{\sigma }_{{a, {\text{int}}}}^{2}$ = genetic variance of DGC at the intercept point; $\widehat{\sigma }_{{e, {\text{int}}}}^{2}$ = residual variance of DGC at the intercept point; $\widehat{\sigma }_{{a, {\text{sl}}}}^{2}$ = genetic variance of the reaction norm slope; $\widehat{\sigma }_{{P, {\text{Total}}}}^{2}$ = total phenotypic variance of DGC across environments; $h_{\text{int}}^{2}$ = heritability of DGC at the intercept $(\widehat{\sigma }_{{a, {\text{int}}}}^{2} /\widehat{\sigma }_{{P, {\text{int}}}}^{2} )$, where $\widehat{\sigma }_{{P, {\text{int}}}}^{2}$ is the phenotypic variance of DGC; co-heritability of TS $\left( {\frac{{6\widehat{\sigma }_{{a, {\text{int}}, {\text{sl}}}} }}{{\widehat{\sigma }_{{a, {\text{int}}}}^{2} + \widehat{\sigma }_{{e, {\text{int}}}}^{2} }}} \right)$, where $\widehat{\sigma }_{{a, {\text{int}}, {\text{sl}}}}$ is the additive genetic covariance between the intercept and slope

$h_{\text{TS}}^{2}$ = heritability of the slope $\left( {\frac{{\widehat{\sigma }_{{a, {\text{sl}}}}^{2} \times \widehat{\sigma }_{a, X}^{2} }}{{\widehat{\sigma }_{{P, {\text{Total}}}}^{2} }}} \right)$, where $\widehat{\sigma }_{{a, {\text{sl}}}}^{2} \times \widehat{\sigma }_{a, X}^{2}$ is the additive genetic variance of the slope multiplied by the variance of environmental values $X$, respectively; $r_{{{\text{G}}({\text{int}}, {\text{sl}})}}$ = genetic correlation between the intercept and slope $\left( {\frac{{\widehat{\sigma }_{{a_{{{\text{int}}, {\text{sl}}}} }} }}{{\sqrt {\widehat{\sigma }_{{a, {\text{int}}}}^{2} \times \widehat{\sigma }_{{a, {\text{sl}}}}^{2} } }}} \right)$

* Estimate that is significantly different from 0 (95% CI does not include zero)

**Fig. 2:**
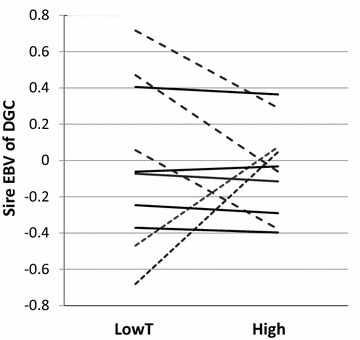
Genetic reaction norms of ten rainbow trout sires across two temperature environments. Ten sires with the highest absolute EBV (five *dashed lines*) and close to zero EBV (five *solid lines*) for the slope are represented. The lines connect the sire EBV for daily growth coefficient (DGC) across the two temperature environments, when the intercept was placed at the lower temperature (*X* = 0)

The genetic correlation between DGC and its TS was significant and strongly negative (−0.64) when the lower temperature environment was used for the intercept (Table 2). This implies that a high DGC at the lower temperature is genetically associated with increased TS across environments. However, Fig. 2 shows that the genotypes (sires) with steep slope EBV were not consistently those that showed the fastest growth potential in the intercept environment. In fact, some genotypes associated with slow growth also showed considerable sensitivity to thermal change, although in the opposite direction. When the intercept was placed at the higher temperature, the estimated genetic correlation was positive (0.38) although not statistically different from zero (based on the large standard error; Table 2). Hence, the growth potential of genotypes at the higher temperature may not show an association with differences in TS when the individuals are moved to the lower temperature.

## Discussion

The temperature difference of 6 °C in our experiment was sufficient to cause substantial variation in the growth reaction norms among 1-year-old rainbow trout families, which indicated heritable differences in their TS. Indeed, the TS of DGC involved a considerable amount of genetic variation, which indicates a potential for response to targeted selection on TS. Both the descriptive parameter $h_{\text{TS}}^{2}$ and the co-heritability, which explain the heritable variance of TS of growth, proved to be moderate. Our finding is not consistent with previous data in the literature, including 18 studies on fish growth traits, which showed that the heritability of macro-environmental sensitivity is generally low [23, 24]. For example, Sae-Lim et al. [23] who used a multigeneration dataset on Finnish rainbow trout did find substantial additive genetic variation in macro-environmental sensitivity of body weight, but the estimated heritability was low (0.07). In their study, the two macro-environments were a freshwater breeding nucleus and a sea test station, which had discrete locations and diverged in several environmental factors, including water salinity and temperature, and rearing system as a whole (earth-bottomed raceways vs. sea net cages, fish density, diet and feeding intensity). Our results support the idea that genetic variation in macro-environmental sensitivity would be smaller than that in the phenotypic value of the trait at the intercept point [24].

As expected, in our study, the rainbow trout showed, on average, lower feed intake, faster growth and better feed conversion ratio at the lower (14 °C) than at the higher rearing temperature (20 °C). The percentage decrease in least square means of DGC was 3.5 from low to high temperature. Under conditions, with a normal oxygen content in the water and a sufficient amount of food provided, the juvenile rainbow trout are known to perform well at temperatures of 14 to 19 °C, the optimum temperature for growth being around 17 °C; temperatures higher than 20 °C cause fast decline in growth rate [15, 16, 26, 27]. Furthermore, in general, feed intake has a higher optimum temperature and feed conversion ratio a lower optimum temperature than growth rate of fish [28]. These facts are likely responsible for the observed differences in feed intake, growth and feed conversion ratio between the experimental temperatures. During the experiment, the water quality parameters that were analyzed i.e. oxygen content, pH and nitrogen compounds were at favorable levels for salmonid aquaculture under both temperature conditions [29–31]. Thus, it is unlikely that other water quality parameters than temperature affected the observed results.

Our results show that the estimates of the genetic parameters for DGC were not dramatically influenced by the temperature environments in which the fish were reared. Environmental stress was reported to both increase and decrease the additive genetic variation in important life-history and morphological traits [32, 33]. On the one hand, the amount of environmentally-induced variation may typically increase under unfavorable conditions, and thus decrease the proportion of genetic variation and in turn, the estimated heritability e.g. [34, 35]. On the other hand, the opposite effect was reported in some animal breeding studies; challenged environments increase the genetic variance of a trait more than the residual variance, which results in higher heritability e.g. [36]. In this study, the heritability of DGC (at the intercept) was similar for the two temperatures tested. Both the genetic and residual variance proved to be larger at the lower temperature, which was presumably a milder environment in terms of growth. One can expect that the difference in heritable potential of growth (mean) manifests itself only at more extreme temperatures above or below the optimum.

Multi-trait models have been used in many studies on rainbow trout to show the presence of G × E interactions in growth [37–41]. Multi-trait and random regression models are equivalent when the dimension of the genetic covariance matrix and the fixed effects included in the models are the same [7, 23]. The genetic correlation of DGC between temperature environments was equal to 0.47 (SE 0.20) when a bivariate model was used with our data (see “Appendix”), which confirms the strong re-ranking of families across the two temperatures tested. The same parameter estimate can also be obtained from the random regression model using the genetic (co)variances. Consequently, selection in either of the temperature environments will presumably result in lower-than-expected genetic gains in the other environment, if the G × E interaction is not taken into account [41–43]. Although the genetic correlation that is estimated from a multi-trait model does express the degree of re-ranking among families, it does not describe how macro-environmental sensitivity of the trait can actually evolve across environments. One advantage of the random regression model is that the genetic parameters for macro-environmental sensitivity of a given trait can be obtained directly, which allows implementation of macro-environmental sensitivity as a trait in the selection index [5, 12, 23]. Yet, an assumption for this in our study is that the slopes, estimated from the two environment points, are linear.

Environmental sensitivity was generally shown to increase in response to selection for high phenotypic values when G × E interaction is present [5, 6, 44]. According to Jinks and Connolly [45], this should be true especially when selection for a high phenotypic value occurs in an environment that produces a phenotype with a higher value compared to another environment (synergistic selection). Correspondingly, selection for decreased environmental sensitivity may generally result in reduced mean productivity in more favorable environments [46]. In this study, the genetic correlation between DGC (at the intercept) and its TS (slope) was markedly negative, when the intercept was placed at the lower temperature. This finding is consistent with the assumption of Jinks and Connolly [45]: selection for fast growth in a more favorable temperature environment should favor increased sensitivity, that is, genotypes having steep negative slopes in reaction norms. However, genetically, the most sensitive genotypes with the steepest slope EBV may not consistently have higher genetic potential for growth at the lower temperature, compared to the least sensitive genotypes with flat slope EBV (Fig. 2). In fact, some of the genotypes with a slow growth at the lower temperature also seem to exhibit pronounced sensitivity to thermal change, but in the positive direction (i.e., growing faster at the higher temperature). Negative genetic relationships between production traits and TS have also been reported in terrestrial farm animals. For example, in pigs, a genetic correlation of −0.5 between carcass weight and sensitivity to heat stress was reported [47]. In dairy cattle, genetic correlations between milk yield and heat tolerance ranged from −0.30 to −0.45 [48, 49], whereas, in sheep, this genetic correlation was equal to −0.8 [50]. The observed negative genetic correlation between DGC and its TS and the presence of strong G × E interaction suggest that selection decisions should be based on more than one thermal environment only. However, by applying a restricted selection criterion, the appropriate index weights that produce the desired genetic responses in both growth rate and its TS can be obtained [51]. It would then be possible to simultaneously improve the growth rate across environments and constrain the genetic change in TS.

In addition to the negative genetic correlation between DGC and its TS, the estimated co-heritability for TS was also negative when a lower temperature was used as the intercept environment. Co-heritability is an approximate measure of the inheritance of the association between DGC and its TS, when the selection criterion is DGC in one temperature environment. The co-heritability has the same sign as the correlated response to direct selection, and, unlike $h_{\text{TS}}^{2}$ and genetic correlation, this parameter also reflects the accuracy of selection [52, 53]. Our results are in line with a recent survey on aquaculture studies by Sae-Lim et al. [23], which showed that the growth of rainbow trout in one environment is genetically related to macro-environmental sensitivity across environments. However, the correlated response of TS is less pronounced if selection for improved growth rate occurs at the higher temperature. In this case, the estimated co-heritability was also associated with relatively large standard errors, which suggests that it should be treated with caution. The magnitude of the co-heritability generally increases, irrespective of its sign, with an increase in G × E interaction [23].

In this study, macro-environmental sensitivity of each genotype (fish family) was defined as the difference in DGC between two temperatures. The reaction norm slope is not an individual measure, but its breeding value can only be estimated based on the growth records of relatives in two (macro-)environments. Because the macro-environmental sensitivity is basically a progeny trait, the accuracy of selection is actually higher for the parents than for their offspring (which are used as breeding candidates). Rainbow trout, as many other aquaculture species, produces large families, which effectively contribute to the genetic analysis of macro-environmental sensitivity. Since the co-heritability of TS proved to be moderate, translating this into moderate accuracy of selection, very large family sizes are not required to reach moderate-to-high precision in slope EBV.

For a rainbow trout breeder, a stock performing well across multiple temperatures is the most desirable outcome. The least sensitive families with flat slopes of reaction norms could be selected when the breeding goal is to obtain robust fish that thrive under variable temperature conditions (increased stability). Alternatively, families with positive growth responses at higher (or lower) temperatures can be chosen when developing a locally-adapted population for a certain environment. In the latter case, TS can be viewed rather as an advantageous character to be used by selective breeding when improving the ‘fit’ between the selected fish and the thermal environment in which they are reared [54]. Either way, it is likely that there will be a high demand for more heat-tolerant populations of rainbow trout in the future since temperatures will continue to increase around the world due to global warming.

This study was undertaken as part of a capacity building project with the Research Institute for Aquaculture No. 1 (RIA-1) in Northern Vietnam where a national breeding program for rainbow trout was recently established from the Finnish broodstock (Research Center for Cold Water Aquaculture Species, RIA-1). Vietnam is estimated to be one of the world’s most vulnerable areas for the negative impacts due to climate change. This is the case, in particular, with cold water aquaculture, for which temperature and water availability are the main limiting factors [55]. The fish used in this experiment shared relatedness with the RIA-1’s broodstock. Combining our results with the records of fish performance in Vietnam under higher temperatures will enable the assessment of selective breeding possibilities in order to decrease TS in the next breeding generations. This will aid in expanding rainbow trout production to lower latitudes and altitudes with better and more stable water resources, and the fish farming sector to adapt to climate change.

## Conclusions

We found that the 1-year-old rainbow trout exhibit substantial genetic variation in growth responses across different rearing temperatures. In terms of growth and feed conversion efficiency, the fish performed better predominantly under the lower (14 °C) than the higher temperature conditions (20 °C). Owing to large additive genetic variation, permanent changes in TS of growth are possible in the studied population. There is a trade-off between growth rate and its TS, since strong selection for faster growth at the lower and more favorable temperature will presumably result in less temperature-tolerant fish. However, the correlated genetic change in TS could be effectively controlled while selecting for high growth across environments, especially if slope EBV are incorporated into the selection index with appropriate weighting.

## Appendix

In a multi-trait model, a trait that is recorded in different environments is treated as separate traits (see Additional file 4). Then, the magnitude of the re-ranking of the genetic groups can be quantified by calculating the genetic correlation (*r*_G_) of the trait in each pair of the environments [42, 56]. A multi-trait animal mixed model was used in this study and run using ASReml 3.0. The bivariate model was as follows:

$$y_{ij} = \mu + a_{i} + e_{ij} ,$$

where *y*_*ij*_ is the observation of the *i*th individual for the *k*th trait (DGC being recorded in two thermal environments), *μ* is an overall trait mean, *a*_*i*_ *i*s the random additive genetic effect of the *i*th individual, and *e*_*ij*_ is the random residual effect. It was assumed that the random variable *a* is multi-normally distributed with a mean of 0 and variance ${\mathbf{A}} \otimes {\mathbf{G}}$, where ${\mathbf{A}}$ is the additive genetic relationship matrix derived from the pedigree traced back to the base population and ${\mathbf{G}}$ is the additive genetic (co)variances matrix. In this study:

$${\mathbf{G}} = \left[ {\begin{array}{*{20}c} {\sigma_{{a,{\text{lowT}}}}^{2} } & {\sigma_{{a,{\text{lowT}}, a, {\text{highT}}}} } \\ {\sigma_{{a, {\text{lowT}}, a, {\text{highT}}}} } & {\sigma_{{a, {\text{highT}}}}^{2} } \\ \end{array} } \right],$$

where $\sigma_{{a,{\text{lowT}}}}^{2}$ and $\sigma_{{a, {\text{highT}}}}^{2}$ are the additive genetic variances of DGC measured at low and high temperatures, respectively, and $\sigma_{{a,{\text{lowT}}, a, {\text{highT}}}}$ is the additive genetic covariance between low and high temperatures. The residual (co)variance matrix is:

$${\mathbf{R}} = \left[ {\begin{array}{*{20}c} {\sigma_{{e, {\text{lowT}}}}^{2} } & 0 \\ 0 & {\sigma_{{e, {\text{highT}}}}^{2} } \\ \end{array} } \right],$$

where $\sigma_{{e, {\text{lowT}}}}^{2}$ and $\sigma_{{e, {\text{highT}}}}^{2}$ are the residual variances at low and high temperatures, respectively. Because each individual fish was present only in one temperature environment, the residual covariance between temperatures was set to 0.

For both DGC traits, the heritability was calculated as:

$$h^{2} = \sigma_{a}^{2} /\sigma_{P}^{2} ,$$

where $\sigma_{P}^{2}$ is the phenotypic variance (sum of additive genetic and residual variances), i.e. $\sigma_{a}^{2} + \sigma_{e}^{2}$.

The genetic correlation ($r_{\text{G}}$) between DGC measured in two temperature environments was calculated as:

$$r_{\text{G}} = \frac{{\sigma_{{a,{\text{lowT}},a,{\text{highT}}}} }}{{\sqrt {\sigma_{{a,{\text{lowT}}}}^{2} \times \sigma_{{a,{\text{highT}}}}^{2} } }},$$

where $\sigma_{{a, {\text{lowT}}, a, {\text{highT}}}}$ is the covariance between additive genetic values measured at low and high experimental temperatures, and $\sigma_{{a, {\text{lowT}}}}^{2}$ and $\sigma_{{a, {\text{highT}}}}^{2}$ are the additive genetic variances of DGC measured at low and high temperatures, respectively. The genetic parameters estimated using a bivariate animal model are shown in Table 3.

**Table 3:** Genetic parameter estimates (± their approximate standard errors) for DGC in two thermal environments using a bivariate animal model

Parameter	DGC _lowT_	DGC _highT_
$\sigma_{a}^{2}$	0.130 (0.043)	0.190 (0.063)
$\sigma_{e}^{2}$	0.156 (0.028)	0.221 (0.041)
$\sigma_{P}^{2}$	0.286 (0.026)	0.411 (0.038)
*h* ^2^	0.455 (0.120)	0.463 (0.123)
$r_{\text{G}}$	0.468 (0.197)	

## Additional files

**Additional file 1.** Pedigree of the studied rainbow trout.

**Additional file 2.** Individual measurement data for univariate genetic analyses.

**Additional file 3.** Tank mean values for FI and FCR.

**Additional file 4.** Individual measurement data for multivariate genetic analysis.

## References

[1] Berven KA, Gill DE (1983). Interpreting geographic variation in life-history traits. Am Zool.

[2] Via S, Lande R (1985). Genotype-environment interaction and the evolution of phenotypic plasticity. Evolution.

[3] Scheiner SM, Lyman RF (1991). The genetics of phenotypic plasticity. II. Response to selection. J Evol Biol.

[4] Hutchings JA (2011). Old wine in new bottles: reaction norms in salmonid fishes. Heredity (Edinb)..

[5] Kolmodin R, Strandberg E, Jorjani H, Danell B (2003). Selection in the presence of a genotype by environment interaction: response in environmental sensitivity. Anim Sci..

[6] Falconer DS (1990). Selection in different environments: effects on environmental sensitivity (reaction norm) and on mean performance. Genet Res.

[7] De Jong G, Bijma P (2002). Selection and phenotypic plasticity in evolutionary biology and animal breeding. Livest Prod Sci..

[8] Ellen ED, Star L, Uitdehaag K, Brom FWA, Philipsson J, Klopcic M, Reents R (2009). Robustness as a breeding goal and its relation with health, welfare and integrity. Breeding for robustness in cattle.

[9] Rauw WM, Gomez-Raya L (2015). Genotype by environment interaction and breeding for robustness in livestock. Front Genet..

[10] Simms EL (2000). Defining tolerance as a norm of reaction. Evol Ecol.

[11] De Jong G (1990). Quantitative genetics of reaction norms. J Evol Biol.

[12] Sae-Lim P, Gjerde B, Nielsen HM, Mulder H, Kause A (2016). A review of genotype-by-environment interaction and micro-environmental sensitivity in aquaculture species. Rev Aquacult.

[13] Ghalambor CK, McKay JK, Carroll SP, Reznick DN (2007). Adaptive versus non-adaptive phenotypic plasticity and the potential for contemporary adaptation in new environments. Funct Ecol.

[14] Sae-Lim P, Komen H, Kause A, van Arendonk JAM, Barfoot AJ, Martin KE (2012). Defining desired genetic gains for rainbow trout breeding objective using analytic hierarchy process. J Anim Sci.

[15] Jobling M (1981). Temperature tolerance and the final preferendum-rapid methods for the assessment of optimum growth temperatures. J Fish Biol.

[16] Hokanson KEF, Kleiner CF, Thorslund TW (1977). Effects of constant temperatures and diel temperature fluctuations on specific growth and mortality rates and yield of juvenile rainbow trout (*Salmo gairdneri*). J Fish Res Board Can.

[17] Bureau DP, Azevedo PA, Tapia-Salazar M, Cuzon G. Pattern and cost of growth and nutrient deposition in fish and shrimp: Potential implications and applications. In: Cruz-Suárez LE, Ricque-Marie D, Tapia-Salazar M, Olvera-Novoa MA, Civera-Cerecedo R, editors. Avances en Nutrición Acuícola V. Memorias del V Simposium Internacional de Nutrición Acuícola: 19–22 November 2000; Mérida. 2000. p. 112–40.

[18] Kaushik SJ (1998). Nutritional bioenergetics and estimation of waste production in non-salmonids. Aquat Living Resour.

[19] Kenward MG, Roger JH (1997). Small sample inference for fixed effects from restricted maximum likelihood. Biometrics..

[20] Gilmour AR, Gogel BJ, Cullis BR, Thompson R. ASReml user guide release 3.0. Hemel Hempstead: VSN International Ltd; 2009.

[21] Fischer TM, Gilmour AR, Werf JHW (2004). Computing approximate standard errors for genetic parameters derived from random regression models fitted by average information REML. Genet Sel Evol..

[22] Van Tienderen PH, Koelewijn HP (1994). Selection on reaction norms, genetic correlations and constraints. Genet Res.

[23] Sae-Lim P, Mulder H, Gjerde B, Koskinen H, Lillehammer M, Kause A (2015). Genetics of growth reaction norms in farmed rainbow trout. PLoS One.

[24] Scheiner SM (1993). Genetics and evolution of phenotypic plasticity. Annu Rev Ecol Syst.

[25] Kolmodin R, Bijma P (2004). Response to mass selection when the genotype by environment interaction is modelled as a linear reaction norm. Genet Sel Evol..

[26] Wurtsbaugh WA, Davis GE (1977). Effects of temperature and ration level on the growth and food conversion efficiency of rainbow trout. Salmo gairdneri Richardson. J Fish Biol..

[27] Mäkinen T (1994). Effect of temperature and feed ration on energy utilization in large rainbow trout, *Oncorhynchus mykiss* (Walbaum). Aquacult Res..

[28] Jobling M (1994). Fish bioenergetics.

[29] Fivelstad S, Schwartz J, Stromsnes H, Olsen AB (1995). Sublethal Effects and safe levels of ammonia in seawater for Atlantic Salmon postsmolts (*Salmo Salar* L). Aquacult Eng.

[30] Wedemeyer GA, Iwama GK, Pickering AD, Sumpter JP, Schreck CB (1997). Effect of rearing conditions on the health and physiological quality of fish in intensive culture. Fish stress and heath in aquaculture.

[31] Timmons MB, Ebeling JM, Wheaton FW, Summerfelt ST, Vinci BJ. Recirculation aquaculture systems. 2nd Ed. NRAC Publications No. 01-002. Ithaca: Cayuga Aqua Ventures; 2002.

[32] Hoffmann AA, Merilä J (1999). Heritable variation and evolution under favourable and unfavourable conditions. Trends Ecol Evol.

[33] Charmantier A, Garant D (2005). Environmental quality and evolutionary potential: lessons from wild populations. Proc Biol Sci..

[34] Uller T, Olsson M, Ståhlberg F (2002). Variation in heritability of tadpole growth: an experimental analysis. Heredity (Edinb)..

[35] Janhunen M, Piironen J, Peuhkuri N (2010). Parental effects on embryonic viability and growth in Arctic charr *Salvelinus alpinus* at two incubation temperatures. J Fish Biol.

[36] Herrero-Medrano JM, Mathur PK, ten Napel J, Rashidi H, Alexandri P, Knol EF (2015). Estimation of genetic parameters and breeding values across challenged environments to select for robust pigs. J Anim Sci.

[37] Fishback AG, Danzmann RG, Ferguson MM, Gibson JP (2002). Estimates of genetic parameters and genotype by environment interactions for growth traits of rainbow trout (*Oncorhynchus mykiss*) as inferred using molecular pedigrees. Aquaculture.

[38] Kause A, Ritola O, Paananen T, Wahlroos H, Mäntysaari EA (2005). Genetic trends in growth, sexual maturity and skeletal deformations, and rate of inbreeding in a breeding programme for rainbow trout (*Oncorhynchus mykiss*). Aquaculture.

[39] Pierce LR, Palti Y, Silverstein JT, Barrows FT, Hallerman EM, Parsons J (2008). Family growth response to fishmeal and plant-based diets shows genotype × diet interaction in rainbow trout (*Oncorhynchus mykiss*). Aquaculture.

[40] Le Boucher R, Quillet E, Vandeputte M, Lecalvez JM, Goardon L, Chatain B (2011). Plant-based diet in rainbow trout (*Oncorhynchus mykiss* Walbaum): are there genotype-diet interactions for main production traits when fish are fed marine vs. plant-based diets from the first meal?. Aquaculture.

[41] Sae-Lim P, Kause A, Mulder HA, Martin KE, Barfoot AJ, Parsons JE (2013). Genotype-by-environment interaction of growth traits in rainbow trout (*Oncorhynchus mykiss*): a continental scale study. J Anim Sci.

[42] Falconer DS (1952). The problem of environment and selection. Am Nat..

[43] Mulder HA, Bijma P (2005). Effects of genotype x environment interaction on genetic gain in breeding programs. J Anim Sci.

[44] van der Waaij EH (2004). A resource allocation model describing consequences of artificial selection under metabolic stress. J Anim Sci.

[45] Jinks JL, Connolly V (1973). Selection for specific and general response to environmental differences. Heredity.

[46] Rosielle AA, Hamblin J (1981). Theoretical aspects of selection for yield in stress and non-stress environment. Crop Sci.

[47] Zumbach B, Misztal I, Tsuruta S, Sanchez JP, Azain M, Herring W (2008). Genetic components of heat stress in finishing pigs: parameter estimation. J Anim Sci.

[48] Ravagnolo O, Misztal I (2000). Genetic component of heat stress in dairy cattle, parameter estimation. J Dairy Sci.

[49] Aguilar I, Misztal I, Tsuruta S (2009). Genetic components of heat stress for dairy cattle with multiple lactations. J Dairy Sci.

[50] Finocchiaro R, van Kaam JB, Portolano B, Misztal I (2005). Effect of heat stress on production of Mediterranean dairy sheep. J Dairy Sci.

[51] Brascamp E (1984). Selection indices with constraints. Anim Breeding Abstracts.

[52] Falconer DS, Mackay TFC (1996). Introduction to quantitative genetics.

[53] Janssens MJJ (1979). Co-heritability: its relation to correlated response, linkage, and pleiotropy in cases of polygenic inheritance. Euphytica.

[54] Lawrence AB, Wall E (2014). Selection for ‘environmental fit’ from existing domesticated species. Rev Sci Tech.

[55] Institute of Strategy and Policy on Natural Resources and Environment. Viet Nam assessment report on climate change (VARCC). 2009. http://www.unep.org/pdf/dtie/VTN_ASS_REP_CC.pdf. Accessed 27 Sept 2016.

[56] Lynch M, Walsh B (1998). Genetics and analysis of quantitative traits.

